# Corrugator activity confirms immediate negative affect in surprise

**DOI:** 10.3389/fpsyg.2015.00134

**Published:** 2015-02-16

**Authors:** Sascha Topolinski, Fritz Strack

**Affiliations:** ^1^Department of Psychology, Social and Economic Cognition, University of Cologne, Cologne, Germany; ^2^University of Würzburg, Würzburg, Germany

**Keywords:** surprise, fluency, affect, EMG, expectancy

## Abstract

The emotion of surprise entails a complex of immediate responses, such as cognitive interruption, attention allocation to, and more systematic processing of the surprising stimulus. All these processes serve the ultimate function to increase processing depth and thus cognitively master the surprising stimulus. The present account introduces phasic negative affect as the underlying mechanism responsible for this switch in operating mode. Surprising stimuli are schema-discrepant and thus entail cognitive disfluency, which elicits immediate negative affect. This affect in turn works like a phasic cognitive tuning switching the current processing mode from more automatic and heuristic to more systematic and reflective processing. Directly testing the initial elicitation of negative affect by surprising events, the present experiment presented high and low surprising neutral trivia statements to *N* = 28 participants while assessing their spontaneous facial expressions via facial electromyography. High compared to low surprising trivia elicited higher corrugator activity, indicative of negative affect and mental effort, while leaving zygomaticus (positive affect) and frontalis (cultural surprise expression) activity unaffected. Future research shall investigate the mediating role of negative affect in eliciting surprise-related outcomes.

## IMMEDIATE NEGATIVE AFFECT IN SURPRISE

Surprise is a distinct emotional response to events that are discrepant with the schema of a current situation (e.g., Smedslund, 1990; Meyer et al., 1991, 1997; Ekman, 2003). There is no agreement in the literature on the particular valence of surprise as an emotion. While some approaches label surprise as a positive emotion (e.g., Fontaine et al., 2007; Valenzuela et al., 2010), other argue that surprise has no valence at all (e.g., Russell, 1980; Reisenzein and Meyer, 2009; Reisenzein et al., 2012). However, there is growing indirect evidence that particularly the immediate phase of cognitive interruption during surprise triggers negative affect (Noordewier et al., submitted). In several independent lines of research in social psychology it has been shown that inconsistencies, disruption, and lack of structure are experienced as unpleasant (Elliot and Devine, 1994; Kay et al., 2009; Gawronski and Strack, 2012; Proulx et al., 2012; Rutjens et al., 2013). For instance, Mendes et al. (2007) found that targets who disconfirm a certain stereotype schema, such as a Latino with high socio-economic status, or an Asian with a southern accent, are liked less than stereotype-consistent exemplars. More directly demonstrating a negative component in surprise, Noordewier and Breugelmans (2013) examined the spontaneous facial expressions of individuals who were surprised by unexpected turning of events in TV shows. Facial codings of these expressions showed negative overall expressions.

The evolutionary function of surprise is to facilitate cognitive mastering of unexpected events (Plutchik, 1980; Meyer et al., 1991, 1997; Forabosco, 1992; Attardo, 1997). This is realized by a whole *emotion syndrome* (Reisenzein, 2000) of attentional and cognitive mechanisms that all serve an enhanced and more thorough processing of the surprising stimulus: interruption of ongoing mental operations (Meyer et al., 1991; Reisenzein, 2000), behavioral freezing (Scherer et al., 2004), recruitment of executive capacity (Näätänen, 1990), attention-allocation to the surprising event (e.g., Darwin, 1872/1999; Ekman et al., 2002), and increase in effortful processing (e.g., Meyer et al., 1991, 1997; Scherer, 2001; Horstman, 2006). As a consequence, surprising stimuli are more elaborated cognitively (cf., *sensemaking*, Pezzo, 2003; *cognitive mastering*, Attardo, 1997) and more likely remembered at a later time (e.g., Schützwohl, 1998) in comparison to non-surprising stimuli. In a dual-system perspective, surprise is thus an effective functional switch between more automatic and routine processing to more effortful and reflective operations (Strack and Deutsch, 2004), functioning like a *cognitive tuning* from heuristic to systematic processing (Bless, 2001; Schwarz, 2002; Deutsch and Strack, 2008). The question is how this switch operates. Which powerful mechanism enables all these different yet functionally converging processes?

In this paper, we propose phasic negative affect as a mechanism. In the following, we argue that schema-discrepant events evoke cognitive disfluency which in turn triggers negative affect that tunes the cognitive system to more systematic processing. Thus, in contrast to earlier models that hold that schema-discrepancy triggers surprise as an affectively neutral signal directly (e.g., Meyer et al., 1997), we introduce negative affect as a causal mediator between discrepancy and surprise. This procedural account of surprise lends insights from various cognitive and social psychological frameworks and deepens our understanding of the causal architecture of surprise.

### SURPRISE AS COGNITIVE DISFLUENCY

A powerful psychological concept in explaining various phenomena is processing fluency, which is the content-independent speed and efficiency of ongoing mental operations (Reber et al., 2004), such as perceptual (e.g., Reber et al., 1998), or semantic processing (Whittlesea, 1993). High processing fluency elicits positive affect (Winkielman and Cacioppo, 2001), while low processing fluency elicits negative affect and more thorough processing (Alter et al., 2007). There are various ways to manipulate fluency experimentally, such as degrading and enhancing perceptual clarity (Reber et al., 1998), repeated exposure (Moreland and Topolinski, 2010), motor training (Topolinski and Strack, 2009c, 2010; Topolinski, 2010, 2012), or semantic priming (Whittlesea, 1993). Particularly semantic fluency is of particular interest for the case of surprise. Previous research has shown that the fluency of processing the meaning of a stimulus is facilitated when this stimulus occurs in a semantically predictive context (Whittlesea, 1993) or in semantic coherence with other stimuli (Topolinski and Strack, 2008, 2009a,b,d). For instance, a coherent word triple such like SALT DEEP FOAM (implying the common topic SEA) is processed faster than an incoherent word triple like BALL BOOK DREAM (implying no common topic; Topolinski, 2011).

How is surprise connected to fluency? According to the major theories on surprise, a surprising event is defined as a stimulus or stimulus change that is discrepant or inconsistent with the currently activated general schema of the situation (e.g., Smedslund, 1990; Meyer et al., 1991, 1997; Ekman, 2003). This may be the case because the stimulus was not expected or the expectancy of another stimulus or event had been evoked, like a sweet taste when one has expected a sour taste (Teigen and Keren, 2003). However, a conscious expectancy about an occurring event is not necessary for surprise to occur. Any event that is inconsistent with the current active schema will be surprising, even if no conscious expectancy was held. For instance, running into your house neighbor in a foreign airport is strongly surprising, although you have not held conscious expectancies in mind about the likelihoods of meeting certain people on the airport. In terms of semantic processing, surprising events can thus be conceptualized as events that are inconsistent with the currently activated semantic context. Thus, these events elicit semantic disfluency, which has been shown in previous research to evoke certain psychological consequences. In the following we argue that these consequences should also occur for surprising events.

### SURPRISE, DISFLUENCY, AND NEGATIVE AFFECT

While relatively high processing fluency elicits positive affect, low fluency prompts immediate negative feelings (Hajcak and Foti, 2008; Topolinski et al., 2009; Hajcak, 2012). For instance, low compared to high-fluent stimuli are liked less, judged as being false, toxic, or less famous and funny (Topolinski and Reber, 2010a,b; Topolinski and Strack, 2010; Leder et al., 2013; Topolinski, 2014; Topolinski et al., 2014b). Such a phasic negative affect, independent from fluency, has been shown to function like negative mood in *cognitive tuning*, where negative compared to positive affective states inhibit automatic and heuristic processes and induce more systematic and effortful processing (e.g., Bless et al., 1996; Kuhl, 2000; Baumann and Kuhl, 2002; Ruder and Bless, 2003). For instance, phasic negative compared to positive affect, when induced randomly, changing from trial to trial and lasting only for around a second, decreases creative performance (Topolinski and Deutsch, 2012), or inhibits automatic semantic processing (Topolinski and Deutsch, 2013).

Crucially, also disfluency-triggered negative affect can function as a rapid cognitive tuner to more systematic processing. Alter et al. (2007) showed that experimentally induced disfluency facilitated systematic processing of syllogistic reasoning problems, a core facility of reflective processing (Strack and Deutsch, 2004).

Applying this to surprising events, we argue that surprising stimuli are cognitively disfluent (since they are schema-discrepant by definition) and thereby elicit immediate negative affect that then tunes cognitive processing from a more heuristic, impulsive mode to a more systematic, effortful mode in the further course of psychological functioning. It should be emphasized, however, that this is a subtle negative state that is not necessarily conscious (cf., Russell, 2003; Winkielman and Berridge, 2004; Topolinski and Deutsch, 2012) and not a strong emotional experience. Thus, we do not argue that this brief negative affect determines the eventual experiential evaluation once the surprising event is fully mastered. Of course, we can be positively surprised and feel joy in response to unanticipated positive outcomes, or can be negatively surprised by bad news. This eventual valence of the later surprise feeling is rather determined by the valence of the event itself (see also Noordewier and Breugelmans, 2013). Negative affect only pertains to the immediate state when encountering a schema-discrepant event.

In sum, we argue that negative affect is the mechanism triggering a switch in operation mode from automatic or heuristic processing to more controlled and effortful processing, as well as all further attentional and motivational consequences such a switch from heuristic to analytical processing brings along, such as attention allocation and deeper processing (Meyer et al., 1991; Reisenzein, 2000).

The crucial first prediction of this account, however, is that surprising stimuli elicit negative affect. As already mentioned, this has been shown recently by Noordewier and Breugelmans (2013) for facial codings of TV show participants. However, these expressions were coded as whole-face responses and did not differentiate specific facial muscles indicative of more specific affective responses. Because of this shortcoming, the present study examined affective facial responses to surprising stimuli in a more controlled experimental set-up with a psycho-physiological method, namely facial electromyography (EMG). This also allowed to disentangle independent changes in positive and negative affect (Winkielman and Cacioppo, 2001).

### AIM OF THE PRESENT RESEARCH

The present study tested the initial stage of affective consequences of surprise as outlined above, the automatic elicitation of negative affect. Note that the present scope was not about the further cognitive consequences of surprise such as attentional allocation or deeper processing. The current notion of (dis)fluency only refers to the immediate online efficiency in encoding a stimulus, not the later increased mental effort that is elicited by surprise. As one operationalization of schema-discrepant, thus surprising, information, we chose trivia statements that had been pre-rated as being more or less surprising (Reisenzein, 2000). For the case of trivia, the surprise and thus disfluency does not stem from a situationally primed context or even expectancy, but from the degree to which certain trivia are (in)consistent with our chronically activated *general knowledge structures* (e.g., Bless et al., 1996). Thus, surprising trivia do not match semantic knowledge structure and thus exhibit *semantic disfluency*.

As a genuine indicator of spontaneous affect we assessed spontaneous facial muscle activity via facial EMG (Cacioppo et al., 1986; Dimberg et al., 2000; but see, for recent debate on the coherence between affect and facial expression, Hassin et al., 2013; Reisenzein et al., 2013). Specifically, three muscles were investigated that have been shown to be indicative of affective responses. The *M. zygomaticus major*, which raises the corner of the lips in smiling, is indicative of positive affect (Cacioppo et al., 1986; Scherer and Ellgring, 2007). The muscle has also been shown to be associated with gains in processing fluency (Harmon-Jones and Allen, 2001; Winkielman and Cacioppo, 2001), and with semantic fluency. Topolinski et al. (2009) presented word triples to participants who were told that these would be random words and were asked to merely read over these triples. In some of these triples, the words were not random but semantically coherent (e.g., DEEP FOAM SALT all related to the common concept of SEA), while in other triples the words were actually random, that is, semantically incoherent (e.g., DREAM BALL BOOK). It was found that participants showed higher zygomaticus activity for coherent than for random triples. This occurred because coherent compared to incoherent triples were encoded with a higher semantic fluency.

The *M. corrugator supercilii*, which furrows the brows, is indicative of negative affective states (e.g., Ekman, 1973; Cacioppo et al., 1986) and to difficulty in information processing, that is, disfluency (Cacioppo et al., 1985; Cohen et al., 1992; see, for inductions of subjective mental effort due to corrugator activation, Larsen et al., 1992; Stepper and Strack, 1993; Strack and Neumann, 2000). In Topolinski et al. (2009), higher corrugator activity was found during encoding of incoherent compared to coherent semantic information. This makes the corrugator the prime indicator of disfluency-triggered negative affect in the present argumentation.

Finally, the *M. frontalis medialis*, which raises the eye brows, surely is the prima facie indicator of surprise, since the iconic surprise face in cultural displays and actors involves raised eye brows (Scherer and Ellgring, 2007). And indeed, Topolinski et al. (2009) found increased activity of the frontalis for the encoding of incoherent (i.e., disfluent) semantic information compared to coherent information. However, several other studies that instantiated real surprising events in more ecologically valid ways, such as changing a whole room, found only a weak impact on frontalis activity (Reisenzein et al., 2006; Schützwohl and Reisenzein, 2012).

Following the current argumentation that surprise prompts negative affect, we predicted increased corrugator activity for highly compared to low surprising trivia statements (cf., Topolinski and Strack, 2009b). Furthermore, we predicted lower zygomaticus activity for highly compared to low surprising stimuli as disfluent surprising stimuli should elicit negative affect (cf., Winkielman and Cacioppo, 2001; Topolinski et al., 2009). Concerning the frontalis, it remained an empirical question whether frontalis would be susceptible to the present manipulation (see Topolinski et al., 2009 vs. Reisenzein et al., 2006). We did not implement any other measure (such as explicit affective ratings) to ensure that participants’ facial responses reflected spontaneous affective processes running independent from a conscious evaluative mind-set or any demand effects.

## MATERIALS AND METHODS

### PARTICIPANTS

Twenty-eight volunteers from various professional backgrounds from the city area of Würzburg participated for a financial compensation of €10 (21 female, 7 male, *M*_age_ = 26, SD_age_ = 5). They were recruited via a local mini-job online market. The volunteers were not screened for psychological or neurological disorders, and due to anonymity issues, we did not record other demographic variables such as ethnicity. Unfortunately, this sample size confines the power of the present tests, but it is common for such time-consuming and laborious methods (cf., Winkielman and Cacioppo, 2001).

### MATERIALS

Trivia statements were developed using the pool of quiz items used in Reisenzein (2000).^1^ These original items were compounds of questions with true and false answers. For instance, *The invention of matches is attributed to?*, *Johnny Walker* (correct answer), and *Robert Bosch* (false answer). For these items, norming ratings are available on how surprising the correct answer is to German samples. In these norming ratings, *N* = 60 participants had rated how surprising the given fact was to them. Using these ratings, 22 high-surprising (*M*_surprising_rating_ = 5.67, SD = 0.45) and 22 low-surprising (*M*_surprising_rating_ = 1.80, SD = 0.66) facts were chosen, with a strong resulting difference in normative ratings on surprise in Reisenzein (2000), *t*(42) = 22.68, *p* < 0.001. Then, a trivia statement was created by combining the initial question with the correct answer, for instance *The invention of matches is attributed to Johnny Walker* (highly surprising), or *Women have a higher life expectancy than men* (low surprising). The stimuli (in German language) are available from the first author upon request.

#### Manipulation check

To check whether these materials still elicited similar surprise levels than in the rating by Reisenzein (2000), *N* = 12 individuals similar in age and education to the main sample and being ignorant about the current research thrust received these 44 items and were asked to rate how surprising each item is (0 = *not at all surprising* to 10 = *very surprising*). As expected given the selection of extreme items, there was a very large difference between the items: (*M*_high_surprise_items_ = 6.97, SD = 1.00 vs. *M*_low_surprise_items_ = 2.10, SD = 0.90), *t*(11) = 15.17, *p* < 0.001, *d* = 5.23.

### PROCEDURE

The study had ethical approval from the Deutsche Forschungsgemeinschaft (Str 264/25-1). After informed consent, participants were tested in single sessions. Participants were told that skin conductance was measured by electrodes to cover the actual facial muscle recording (see Dimberg et al., 2000). The trivia viewing task was part of a larger experimental battery involving other, unrelated tasks (studying non-sense words, Topolinski, 2012; watching neutral geometric shapes, Topolinski et al., submitted). In the trivia viewing task, participants were told that a recreation phase in between two other tasks, necessary for physio-technological reasons, would follow in which participants should relax. For their entertainment, general trivia facts would be presented on the PC screen. They were not given any specific encoding instruction but were asked to stay relaxed and watch the events unfolding on the PC screen. Thus, participants simply read the trivia statements, and no response or any task was required. Then, in altogether 44 trials, 22 high surprising and 22 low surprising trivia items were presented in random order, re-randomized anew for each participant. In each trial, first a fixation cross appeared in the middle of the screen for 1000 ms followed by a blank screen for 1100 ms. Then the trivia item was presented in black font in the middle of screen for 6000 ms. Then, an inter-trial interval being a white screen followed with a length randomly varying between 5000 and 6000 ms. The whole task took around 10 min.

### EMG ASSESSMENT

The electrical activity was measured over the *M. zygomaticus major*, the *M. corrugator supercilii*, and the *M. frontalis medialis* on the left side of the face using bipolar placements of 13/7 mm Ag/AgCl surface-electrodes (Fridlund and Cacioppo, 1986). The impedances of all electrodes were reduced to less than 10 kOhm. The EMG raw signal was measured with a V-Amp amplifier (Brain Products Inc.), digitized by a 24-bit analog-to-digital converter, and then stored on a computer with a sampling frequency of 500 Hz. These raw data were rectified offline and filtered with a 30 Hz low cutoff filter, a 500 Hz high cutoff filter, a 50 Hz notch filter, and a 125 ms moving average filter.

To control for baseline activity, EMG scores were calculated being the difference between the activity in the given trial and a pre-stimulus level, namely the mean activity during the last 1000 ms before stimulus onset. Trials with an EMG activity above 8 μV during the baseline period and above 30 μV during the stimuli presentation were excluded (<8%). Then, the EMG scores were aggregated over the 6000 ms of trivia item presentation, for high and for low surprising trivia, respectively.

## RESULTS

**FIGURE 1:**
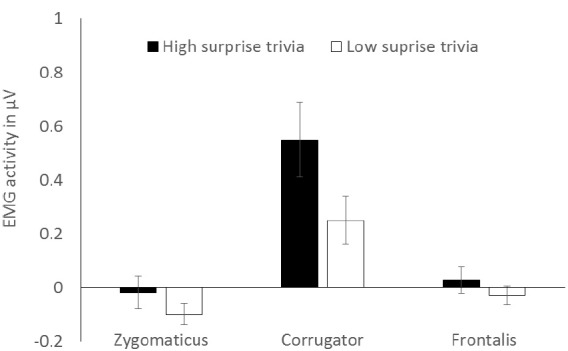
**EMG activity as a function of surprise and muscle.** Error bars indicate ±1 SEM.

The conditional means are shown in Figure 1. A 3 (muscle: zygomaticus, corrugator, frontalis; within) × 2 (trivia: high surprise, low surprise; within) analysis of variance (ANOVA) over the electrodermal activity yielded a main effect of muscle, *F*(1,26) = 6.94, *p* = 0.004, ${\text{η}}_{\text{p}}^{\text{2}}$ = 0.35, with generally higher activity of the corrugator compared to the other muscles overall, which is conceptually irrelevant. Furthermore, a main effect of trivia surfaced, *F*(1,27) = 6.04, *p* = 0.021, ${\text{η}}_{\text{p}}^{\text{2}}$ = 0.19, with generally more muscle activity for surprising than for common trivia. Crucially, a marginal interaction between muscle and trivia was found, *F*(1,26) = 3.15, *p* = 0.060, ${\text{η}}_{\text{p}}^{\text{2}}$ = 0.20.

According to our predictions of a differential impact of surprise on the single muscles, we ran single planned tests separately for each muscle. For the corrugator, there was higher activity during encoding of high surprising compared to low surprising trivia, *t*(27) = 2.61, *p* = 0.014, *d* = 0.48, 95% CI [0.06, 0.53]. There were no effects of surprise on zygomaticus and frontalis activity (both *t*s < 1.4, *p*s > 0.19), see also Figure 1. Corrugator activity was reliably above zero for both high and low surprising items (*t*s < 3.91, *p*s < 0.011). Zygomaticus activity was reliably below zero for less surprising items, *t*(27) = 2.61, *p* = 0.015.

## DISCUSSION

Examining the initial affective responses to neutral surprising stimuli, we assessed spontaneous facial expressions during merely reading high and low surprising trivia statements. As we had argued, high compared to low trivia statements are inconsistent with the individuals’ common knowledge structures (Bless et al., 1996) and are thus cognitively disfluent, eliciting negative affect (Alter et al., 2007; Topolinski et al., 2009; Noordewier and Breugelmans, 2013). We found increased corrugator activity for high surprising compared to low surprising trivia, while zygomaticus and frontalis were not significantly affected. Before discussing this result, it should be emphasized that the present rather small sample confined the present statistical power, and the present preliminary evidence should be interpreted with caution.

This finding corroborates our assertation that surprise entails an immediate negative affective state and provides more specific evidence on facial expressions than whole-face codings (see also Noordewier and Breugelmans, 2013). However, the present initial demonstration is only a starting point for experimentally manipulating and testing the whole procedural chain that we assume for surprise to be at work, and also to test the mediational role of negative affect. The current evidence also allows the reversed causal interpretation that negative affect is only a by-product of the whole attentional and cognitive syndrome of surprise of increased cognitive effort and cognitive mastering (Topolinski, 2014). Future studies should show that high compared to low surprising events do not only elicit negative affect, but that this negative affect in turn induces a more thorough processing of surprising stimuli and is thus correlated with further psychological consequences of surprise, such as attention allocation and more reflective processing (Plutchik, 1980; Schützwohl, 1998; Reisenzein, 2000). Furthermore, negative affect in the present set-up might have stemmed not only from the initial dysfluency in reading the trivia, but from frustration in additional stimulus elaboration and memory-retrieval during failure of making sense particularly of surprising items (cf., Pezzo, 2003). However, note that the present time window of EMG measures was only the first 6 s after stimulus onset. Because (1) participants needed 1–2 s to read the trivia in the first place, (2) memory retrieval and fact-checking itself requires another 1–2 s (Collins and Quillian, 1969), and (3) facial activity in response to higher mental processes requires itself time to unfold, we argue that such additional cognitive processes unlikely affected the current data.

A further limitation of the present evidence might be the use of the corrugator muscle as an indicator of negative affect, because the corrugator does not only mark negativity, but also mental effort (Cacioppo et al., 1985; Cohen et al., 1992). It could be argued that the presently found increased activity of the corrugator muscle is rather due to higher mental effort or increased processing depth in integrating the surprising compared to the less surprising trivia statements. However, note that the frontalis muscle was not affected by the present manipulation. In the literature examining the facial responses to mental effort more generally, whenever both corrugator and frontalis were assessed, the frontalis muscle was affected by mental effort to a comparable degree as the corrugator muscle (e.g., Van Boxtel and Jessurun, 1993; Waterink and Van Boxtel, 1994; Waersted and Westgaard, 1996; Bansevicius et al., 1997). Accordingly, the frontalis has been conceptualized as an independent indicator of mental effort (Van Boxtel and Ven, 1978; Fridlund and Cacioppo, 1986). In ergonomics, some authors even suggested frontalis being the more valid indicator of mental effort because it is, in contrast to the corrugator, not affected by valence (Zeier, 1979; de Waard, 1996; Piechulla et al., 2003). The impact of the present manipulation on corrugator but not on frontalis activity thus favors our interpretation that negative affect, but not mental effort *per se*, drove the present responses. However, convergent validity of this facial measure should be obtained in future studies using other measures of negative affect, such as evaluations or approach–avoidance movements (e.g., Topolinski et al., 2014c). Also, although the present item pool was already successfully used in earlier publications (e.g., Reisenzein, 2000), it is still possible that the present items elicited not only surprise, but also confusion or annoyance, which should be disentangled in future studies.^2^

The missing impact of surprise on frontalis activity, which is the prima facie muscle for iconic cultural displays of surprise (for a discussion, see Noordewier and Breugelmans, 2013), might be regarded as being at odds with earlier findings that frontalis is reliably affected by highly surprising events (e.g., Reisenzein et al., 2006). However, detectable frontalis activity in these earlier studies was either weak or occurred in only up to a third of the observed participants (Reisenzein, 2000; Reisenzein et al., 2006; Schützwohl and Reisenzein, 2012). Given that the trivia items we used were not validated in the present participant pool, it could well be possible that they did not elicit enough surprise to trigger frontalis activity (which is weak as it is). Future research is necessary to further ascertain the role of frontalis in the display of surprise.

The null-finding on zygomaticus activity was not predicted and deserves some discussion. In contrast to earlier findings that high compared to low fluency increases zygomaticus activity, thus induced positive affect independent from changes in negative affect (Winkielman and Cacioppo, 2001; Topolinski et al., 2009) we found no impact of surprise on zygomaticus activity. The first possible explanation of course is lacking power, since the present sample size was small (but similar to earlier studies in this domain, e.g., Winkielman and Cacioppo, 2001). However, the direction of the difference in zygomaticus activity was, descriptively, even opposite to what would be expected on fluency grounds: participants exhibited descriptively *higher* zygomaticus activity for high- than low-surprising items.

This can be explained by the relativity of fluency effects (Hansen et al., 2008; Hansen and Topolinski, 2011), as revealed by a closer comparison between the earlier EMG study on semantic fluency (Topolinski et al., 2009) and the present study. In Topolinski et al. (2009), participants received coherent and incoherent word triples and were told that these word triples were random. In addition to this information, one would usually not expect a regularity in word groups in general. Thus, coherent triples showed an unusual fluency gain, higher than one would expect when reading random word groups, eliciting a positive affect (as evidenced by increased zygomaticus activity, but also higher liking ratings, Topolinski and Strack, 2009a,d). In the present study, however, participants received high and low surprising trivia statements. Here, low surprising trivia, although of course having higher semantic fluency than highly surprising trivia, did not exhibit an usually high fluency *compared to what one would expect during reading trivia statements in general*. Thus, an unusual fluency gain and thus positive affect were less likely to occur in the present set-up. However, future research should more closely investigate the respective impact of semantic fluency on positive and negative facial responses under different fluency expectations. Finally, it is also possible that the present trivia statements were simply more complex than the word triples in Topolinski et al. (2009), imposing higher cognitive demand on participants and thereby inhibiting zygomaticus activity more generally.

Finally, a conceptual integration between the present account and earlier highly influential accounts on the mechanism of surprise should be made. While we completely concur with most aspects of the model provided by Meyer and Reisenzein (e.g., Meyer et al., 1991; Reisenzein, 2000), the difference between both accounts is that Meyer et al., (1991) assume that unexpectedness or schema-discrepancy triggers the neutral state of surprise along with all its components directly, while we assume that discrepancy impacts on the cognitive architecture via the link of brief negative affect. We add this mechanistic assumption because we do not know of any plausible perceptual, cognitive, or (other) affective route by which schema-discrepancy should directly causally change the information processing mode (as assumed by Meyer and Reisenzein). Given the previous independent evidence that disfluency triggers negative affect and modulates processing style (e.g., Winkielman and Cacioppo, 2001; Alter et al., 2007), negative affect is an obvious candidate to effectuate such a modulation; and the detection of brief negative affect in the present experiment is first supporting evidence for our claim. Furthermore, if unexpectedness directly triggers a change in processing mode without any further mediator (like in the model by Meyer and Reisenzein), the question is why immediate brief negative affect did occur in the present study as well as in Noordewier and Breugelmans (2013). Thus, we deem the assumption that schema-discrepancy modulates the cognitive operational mode by triggering a brief negative affect as the most parsimonious interpretation and integrative interpretation of the present and earlier findings.

Concluding, the present evidence showed immediate negative facial responses to neutral stimuli that were more or less surprising. Future research should map the mediating role of negative affect in eliciting further cognitive consequences of surprise.

### Conflict of Interest Statement

The authors declare that the research was conducted in the absence of any commercial or financial relationships that could be construed as a potential conflict of interest.

## Footnotes

1 We thank Rainer Reisenzein for kindly supplying the whole stimulus pool and norming ratings.

2 We thank the reviewer for these thoughts.
